# Predictive Values of Pathological and Clinical Risk Factors for Positivity of Sentinel Lymph Node Biopsy in Thin Melanoma: A Systematic Review and Meta-Analysis

**DOI:** 10.3389/fonc.2022.817510

**Published:** 2022-01-27

**Authors:** Hanzi Huang, Ziyao Fu, Jiang Ji, Jiuzuo Huang, Xiao Long

**Affiliations:** Department of Plastic Surgery, Peking Union Medical College Hospital (PUMCH), Chinese Academy of Medical Sciences and Peking Union Medical College, Beijing, China

**Keywords:** thin melanoma, sentinel lymph node biopsy, positive rate, ulceration, microsatellites, Breslow thickness, mitosis rate

## Abstract

**Background:**

The indications for sentinel lymph node biopsy (SLNB) for thin melanoma are still unclear. This meta-analysis aims to determine the positive rate of SLNB in thin melanoma and to summarize the predictive value of different high-risk features for positive results of SLNB.

**Methods:**

Four databases were searched for literature on SLNB performed in patients with thin melanoma published between January 2000 and December 2020. The overall positive rate and positive rate of each high-risk feature were calculated and obtained with 95% confidence intervals (CIs). Both unadjusted odds ratios (ORs) and adjusted ORs (AORs) of high-risk features were analyzed. Pooled effects were estimated using random-effects model meta-analyses.

**Results:**

Sixty-six studies reporting 38,844 patients with thin melanoma who underwent SLNB met the inclusion criteria. The pooled positive rate of SLNB was 5.1% [95% confidence interval (CI) 4.9%-5.3%]. Features significantly predicted a positive result of SLNB were thickness≥0.8 mm [AOR 1.94 (95%CI 1.28-2.95); positive rate 7.0% (95%CI 6.0-8.0%)]; ulceration [AOR 3.09 (95%CI 1.75-5.44); positive rate 4.2% (95%CI 1.8-7.2%)]; mitosis rate >0/mm2 [AOR 1.63 (95%CI 1.13-2.36); positive rate 7.7% (95%CI 6.3-9.1%)]; microsatellites [OR 3.8 (95%CI 1.38-10.47); positive rate 16.6% (95%CI 2.4-36.6%)]; and vertical growth phase [OR 2.76 (95%CI 1.72-4.43); positive rate 8.1% (95%CI 6.3-10.1%)].

**Conclusions:**

The overall positive rate of SLNB in thin melanoma was 5.1%. The strongest predictor for SLN positivity identified was microsatellites on unadjusted analysis and ulceration on adjusted analysis. Breslow thickness ≥0.8 mm and mitosis rate >0/mm^2^ both predict SLN positivity in adjusted analysis and increase the positive rate to 7.0% and 7.7%. We suggest patients with thin melanoma with the above high-risk features should be considered for giving an SLNB.

## Introduction

The incidence of melanoma has been increasing rapidly over the past few decades, with 100,350 new cases diagnosed in America in 2020, most of which are thin melanoma (T1, ≤1.0 mm) (1). Although thin melanomas have a relatively good prognosis with a 10-year survival rate of more than 95%, the absolute number of deaths is notable because of the volume of the disease (2).

To identify melanoma with a poor prognosis and provide more precise treatment, sentinel lymph node biopsy (SLNB) was proposed by surgeons. SLNB is generally considered appropriate for melanoma of T2 or thicker, but the indications for sentinel lymph node biopsy for thin melanoma are still controversial. The positive rate of SLNB for thin melanoma reported by previous studies is approximately 5% (3–5). In addition, SLNB carries a false negative rate of 12.5% (6) and several unwanted complications, including infection (2.9%), seroma (5.1%), hematoma (0.5%), lymphoedema (1.3%), and nerve injury (0.3%) (7).

It is critical to recognize thin melanoma with high-risk pathologic features and to reduce unnecessary invasive manipulation. The mainstream view is that SLNB should be performed in thin melanomas only if high-risk features are indicating SLNB positivity and worse prognosis, such as Breslow thickness >0.75 mm, ulceration, Clark level IV/V, and/or high mitotic rate (4, 8). The American Joint Committee on Cancer (AJCC) 8^th^ edition of the guidelines for melanoma published in 2018 is currently in wide clinical use. T1 melanoma was reclassified into T1a (<0.8 mm) and T1b (0.8-1.0 mm, or any ulceration ≤1 mm) (9). According to the National Comprehensive Cancer Network (NCCN) guidelines of cutaneous melanoma, SLNB is recommended for T1b melanoma or T1a lesions with mitosis rate ≥2/mm^2^, lymphovascular invasion, or other combination of risk factors (10). In the European consensus-based interdisciplinary guideline for melanoma, however, SLNB is recommended only for melanoma ≥0.8 mm with ulceration, mitosis rate ≥1/mm^2^, microsatellites, or other risk factors (11).

The purpose of this meta-analysis was to determine the positive rate of SLNB in thin melanoma and to summarize the predictive value of different clinical and high-risk pathological features for positive results of SLNB.

## Methods

This meta-analysis followed and adhered to the Preferred Reporting Items for Systematic Reviews and Meta-Analyses guidelines.

### Search Strategy

We searched literature published between January 2000 and December 2020 from the PubMed, Embase, Web of Science, and Cochrane Library databases. English articles with “melanoma”, or “melanomas”, and “sentinel lymph node biopsy”, or “SLNB”, or “SNB” were screened. Through reviewing the titles and abstracts of the retrieved literature, we selected potentially eligible studies preliminarily and further reviewed the full texts to determine whether they met the inclusion criteria. Two authors (HHZ & FZY) reviewed all literature obtained and examined whether each of them met the inclusion criteria.

To reduce potential bias due to the small sample size, we set the included criteria, which require a sample size for each study to be larger than 50. The inclusion criteria were as follows: including patients with a pathologic diagnosis of thin melanoma (Breslow thickness ≤1.0 mm) in the study; performing SLNB for >50 patients with thin melanoma, and reporting an SLN positivity rate. Reference lists of included articles and related literature were manually searched to complete the deficiency of computer search.

When multiple studies reported overlapping or duplicate patient sources, only the most recent and comprehensive study was included. Studies that did not report negative sentinel lymph nodes (SLNs) or included a single isolated high-risk pathologic feature were excluded. Case reports, literature reviews, commentaries, editorials, letters, and conference abstracts were also excluded.

### Data Extraction and Quality Assessment

The following data were extracted from studies: 1) study information, including first author and publication year; 2) patient characteristics, including the number of SLNBs performed in patients with thin melanoma, clinical feature (primary tumor location), high-risk pathologic features [Breslow thickness, mitosis rate, Clark Level, ulceration, regression, microsatellites, vertical growth phase, tumor-infiltrating lymphocytes (TIL) and lymphovascular invasion (LVI)]; 3) outcomes, including the number of positive SLNs found in patients with thin melanoma and number of patients with thin melanoma reporting both positive SLN and high-risk features; 4) adjusted odds ratio (OR) for each high-risk pathologic feature if available.

Two authors (HHZ & FZY) used the Newcastle Ottawa Scale (NOS) to assess the risk of bias in the included studies. The NOS evaluates literature quality in three aspects: selection, comparability, and outcomes. The maximum score was 9, and a score greater than 6 is considered to indicate a low risk of bias.

### Statistical Analysis

The primary outcome was the positive rate of SLNB in thin melanoma (Breslow thickness ≤ 1.0 mm), and the pooled effect was calculated and obtained with 95% confidence intervals (CIs). Forest plots were constructed to visually represent the results. The secondary outcomes were the predictive value of high-risk pathologic and clinical features for positive results of SLNB. Unadjusted ORs and adjusted ORs were pooled and analyzed using a random-effects model. Additionally, pooled positive rates of SLNB in patients with each pathologic feature were calculated. Heterogeneity among studies was calculated by the I^2^ measure of inconsistency, and an I^2^>50% indicated significant heterogeneity. The presence of publication bias was investigated visually using a funnel plot. Meta-analysis was performed by Stata/MP software (version 16.0 for Windows, StataCorp LLC, College Station, TX77845, USA).

## Results

### Characteristics of Included Studies

**Figure 1:**
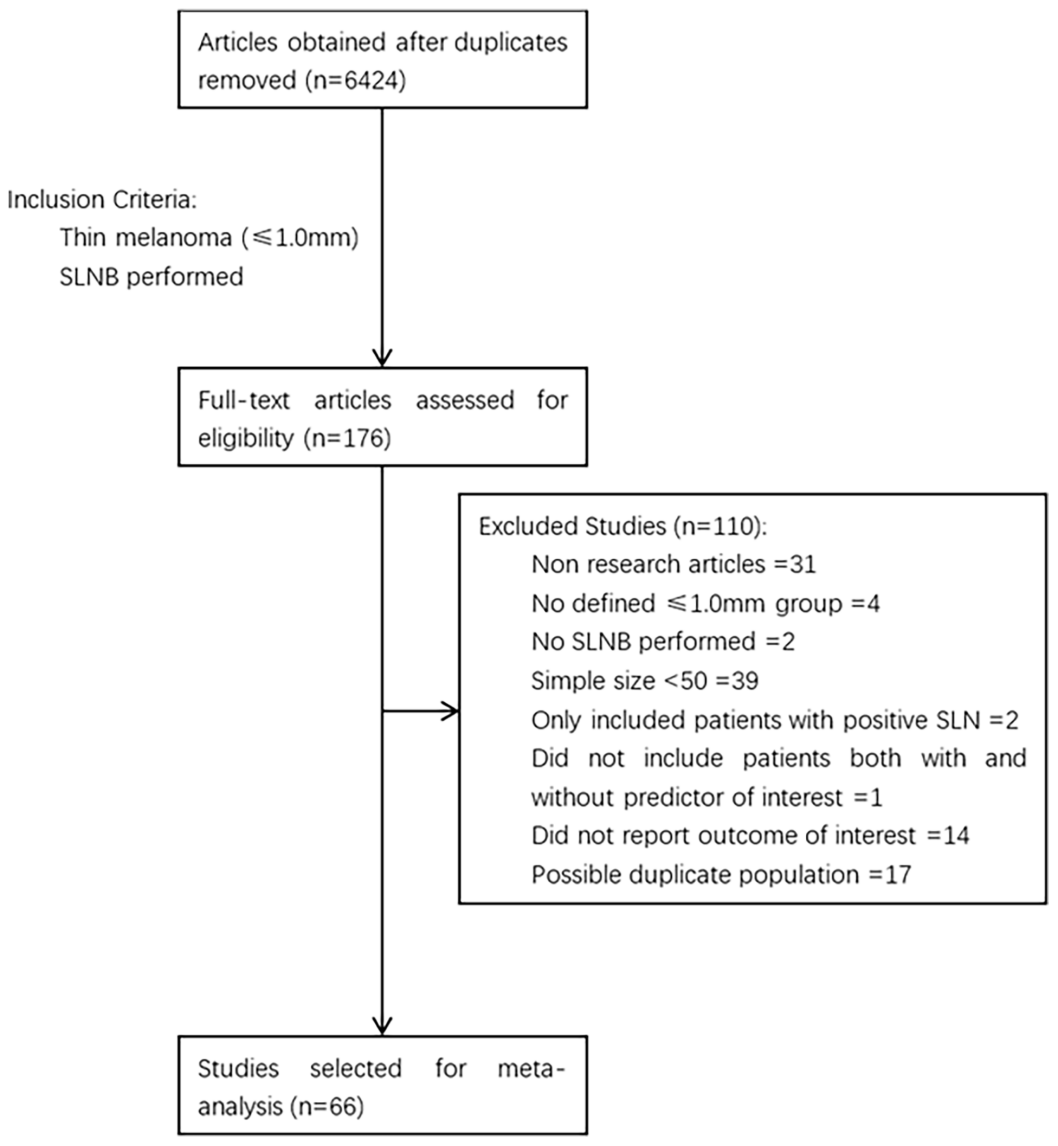
Process study selection.

**Figure 2:**
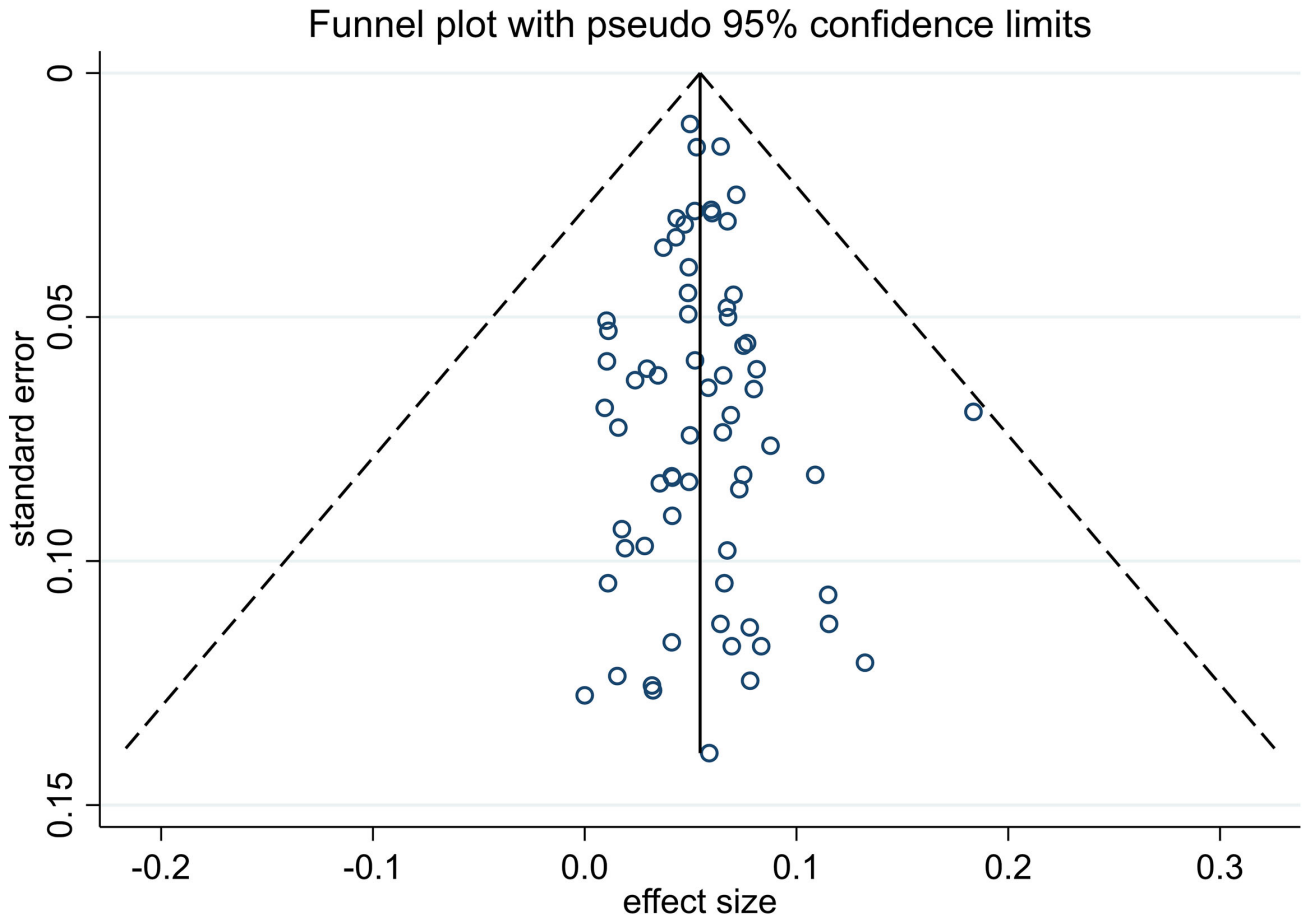
Funnel plot of included studies.

The process of study selection is described in **Figure 1**. A total of 6424 articles were obtained through retrieval, and 66 of them met the inclusion criteria. All of the included studies were retrospective, reporting 38,844 patients with thin melanoma who underwent SLNB (**Table 1**) (8, 12–76). The number of included patients in each study ranged from 51 to 9186, with a median of 205. A total of 2117 (5.45%) positive SLN cases were found among all patients. Thirty-eight of the 66 included studies reported at least one high-risk pathologic feature that may be associated with SLN positivity. A median NOS score of 7 (range from 6 to 8) indicated that the risk of bias of the included studies was small. No study was excluded based on the NOS quality assessment. No significant publication bias among the included studies was found by funnel plot (**Figure 2**).

**Table 1:** Characteristic of the 66 included studies.

Study	Year	Total No. of thin melanoma patients undergoing SLNB	Total No. of thin melanoma patients with positive SLN (%)	High-risk features reported	Risk of bias Score (NOS) (Max=9)
Theile et al. (12)	2020	240	14 (5.8%)	Thickness, ulceration	6
Skochdopole et al. (13)	2020	4332	229 (5.3%)	Thickness	6
Kocsis et al. (14)	2020	78	9 (11.5%)	Ulceration, regression	7
Hu et al. (15)	2020	238	19 (8.0%)	Nil	7
Antonialli et al. (16)	2020	399	27 (6.8%)	Nil	7
Tejera-Vaquerizo et al. (17)	2019	1083	73 (6.7%)	Nil	8
Santos et al. (18)	2019	137	10 (7.3%)	Thickness, ulceration, MR, TIL, regression, CL, microsatellites	8
Piazzalunga et al. (19)	2019	1272	76 (6.0%)	Thickness, ulceration, MR, CL	7
Conic et al. (8)	2019	9186	457 (5.0%)	thickness, ulceration, MR, regression, CL	8
Verver et al. (20)	2018	1607	115 (7.2%)	Nil	7
Stiegel et al. (21)	2018	326	25 (7.7%)	Nil	8
Nguyen et al. (22)	2018	142	7 (4.9%)	Nil	6
Isaksson et al. (23)	2018	1038	49 (4.7%)	Thickness, ulceration, MR	6
Herbert et al. (24)	2018	1129	49 (4.3%)	thickness	7
Tejera-Vaquerizo et al. (25)	2017	203	14 (6.9%)	MR, regression, microsatellites	7
Joyce et al. (26)	2017	65	1 (1.5%)	Thickness, ulceration	8
Wat et al. (27)	2016	171	15 (8.8%)	MR	7
Rubinstein et al. (28)	2016	252	6 (2.4%)	Nil	8
Hieken et al. (29)	2015	4410	283 (6.4%)	Nil	7
Voit et al. (30)	2014	288	15 (5.2%)	Nil	7
Mitteldorf et al. (31)	2014	207	38 (18.4%)	Thickness, ulceration, MR, regression, CL	7.5
Bartlett et al. (32)	2014	781	29 (3.7%)	Thickness, ulceration, MR, TIL, regression, CL, LVI, microsatellites	6.5
Balch et al. (33)	2014	1213	73 (6.0%)	Nil	6
Venna et al. (34)	2013	484	34 (7.0%)	Thickness, ulceration, MR, TIL, CL, LVI	6
van den Broek et al. (35)	2013	61	0 (0.0%)	Nil	6
Mozzillo et al. (36)	2013	492	24 (4.9%)	Ulceration, MR	8
Han et al. (37)	2013	1250	65 (5.2%)	Thickness, ulceration, MR, TIL, regression, CL, LVI, VGP	7.5
Cooper et al. (38)	2013	189	3 (1.6%)	Ulceration, MR, CL	7
Chu et al. (39)	2013	106	3 (2.8%)	Ulceration, MR, CL	8
Ponti et al. (40)	2012	286	3 (1.0%)	Nil	6
Murali et al. (41)	2012	432	29 (6.7%)	Thickness, ulceration, MR, CL, LVI, microsatellites	7
Koshenkov et al. (42)	2012	72	6 (8.3%)	Ulceration, CL	6
Hinz et al. (43)	2012	121	5 (4.1%)	Thickness, ulceration, CL	8
Han et al. (44)	2012	271	22 (8.1%)	Thickness, ulceration, MR, TIL, regression, CL, VGP	7
Elsaesser et al. (45)	2012	212	2 (0.9%)	Nil	7
Yonick et al. (46)	2011	147	16 (10.9%)	Nil	6
Lowe et al. (47)	2011	260	9 (3.5%)	Nil	7
Vermeeren et al. (48)	2010	78	5 (6.4%)	Thickness, ulceration, CL	7
Socrier et al. (49)	2010	68	9 (13.2%)	Regression	6.5
Santillan et al. (50)	2010	72	5 (6.9%)	Nil	7
Mitra et al. (51)	2010	320	24 (7.5%)	Nil	6
Kunte et al. (52)	2010	147	11 (7.5%)	Thickness	7
Ellis et al. (53)	2010	105	2 (1.9%)	Nil	7
Testori et al. (54)	2009	358	4 (1.1%)	Nil	7
Wright et al. (55)	2008	631	31 (4.9%)	Thickness, ulceration, CL	6.5
Roulin et al. (56)	2008	51	3 (5.9%)	CL	7
Kaur et al. (57)	2008	62	2 (3.2%)	Regression	7.5
Starz and Balda (58)	2007	87	10 (11.5%)	Nil	6.5
Koskivuo et al. (59)	2007	141	5 (3.5%)	Nil	7
Vaquerano et al. (60)	2006	91	6 (6.6%)	Nil	7
Ranieri et al. (61)	2006	184	12 (6.5%)	Thickness, ulceration, regression, CL, VGP	7
Nowecki et al. (62)	2006	260	17 (6.5%)	Nil	7
Karakousis et al. (63)	2006	882	38 (4.3%)	Thickness, ulceration, MR, regression, CL, VGP	8
Hershko et al. (64)	2006	64	5 (7.8%)	CL	7
Cascinelli et al. (65)	2006	145	6 (4.1%)	Nil	7
Rex et al. (66)	2005	73	3 (4.1%)	Nil	7
Puleo et al. (67)	2005	409	20 (4.9%)	CL	7
Kesmodel et al. (68)	2005	181	9 (5.0%)	Thickness, ulceration, MR, CL	7
Stitzenberg et al. (69)	2004	146	6 (4.1%)	Ulceration, regression, CL	6
Borgognoni et al. (70)	2004	114	2 (1.8%)	Nil	7
Rousseau et al. (71)	2003	388	4 (1.0%)	Nil	6
Oliveira Filho et al. (72)	2003	77	6 (7.8%)	Ulceration, regression, CL, VGP	7
Jacobs et al. (73)	2003	63	2 (3.2%)	CL	6
Bleicher et al. (74)	2003	272	8 (2.9%)	Thickness	6
Agnese et al. (75)	2003	91	1 (1.1%)	Nil	7
Statius Muller et al. (76)	2001	104	7 (6.7%)	Thickness	7

SLNB, sentinel lymph node biopsy; CL, Clark level; MR, mitotic rate; TIL, tumor-infiltrating lymphocytes; VGP, vertical growth phase; LVI, lymphovascular invasion; PTL, primary tumor location.

### Outcomes

**Figure 3:**
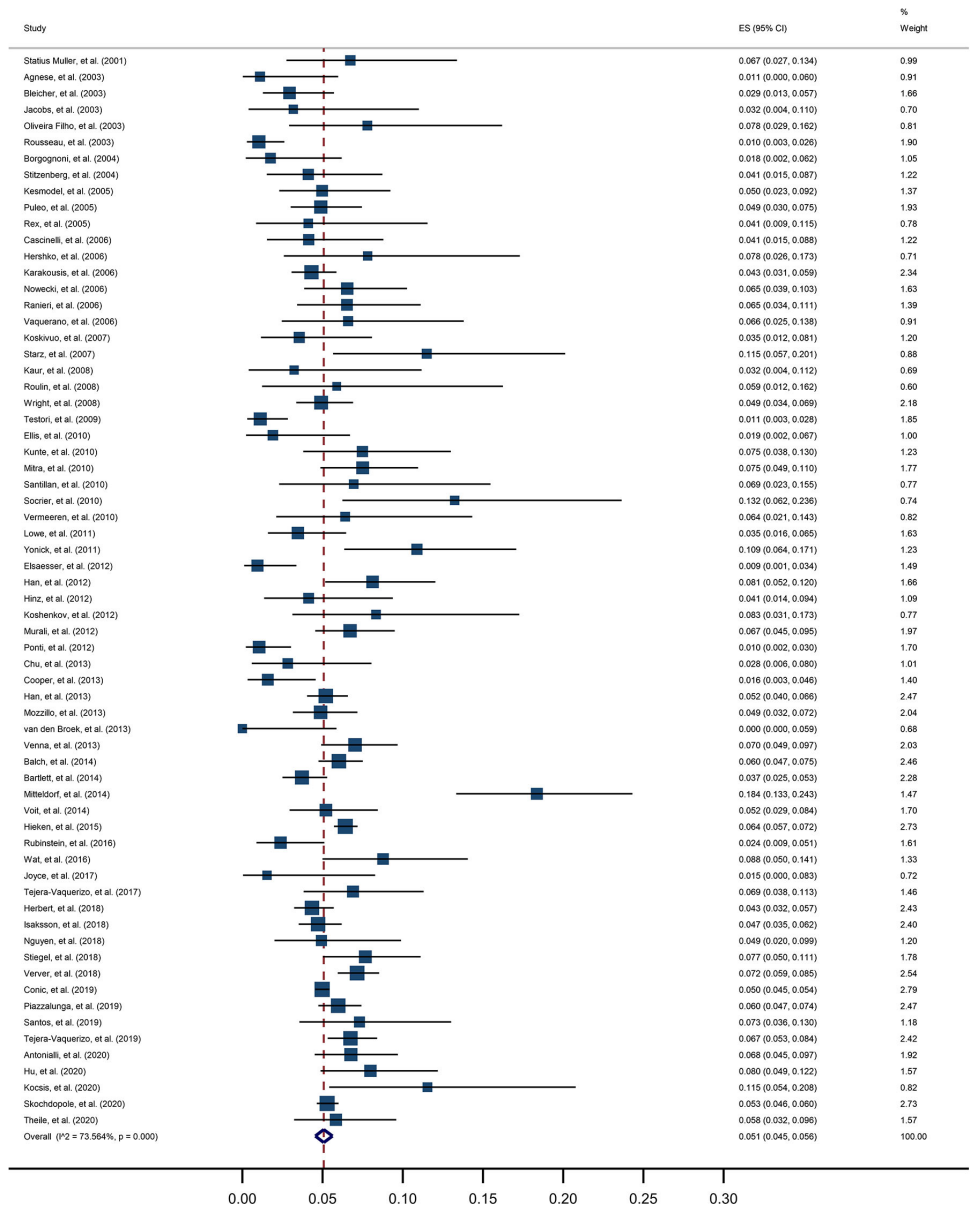
Meta-analysis of sentinel lymph node biopsy positivity in thin melanoma.

For the primary outcome, a pooled positive rate of SLNB was estimated by applying the random effect model, calculated as 5.1% (95% CI, 4.5% to 5.6%, **Figure 3**). Significant heterogeneity between studies was detected (I^2 =^ 73.6%, p<0.001). The unadjusted ORs and pooled positive rate of each high-risk pathologic and clinical feature for SLN positivity is shown in **Table 2**. Breslow thickness ≥0.8 mm, presence of ulceration, mitosis rate >0/mm^2^, Clark level IV/V, and vertical growth phase showed a significant association with SLN positivity in unadjusted analysis. All of the above pathologic features showed a pooled positive rate higher than 5.1% except for the presence of ulceration. Notably, we found the presence of microsatellites to be most strongly associated with SLN positivity, with an unadjusted OR of 3.8 (95% CI, 1.38 to 10.47) and a pooled positive rate of 16.6% (95% CI, 2.4% to 36.6%).

**Table 2:** Predictive value of high-risk pathologic and clinical features for sentinel lymph node biopsy positivity.

Predictor	No. of studies	No. of thin melanoma patients undergoing SLNB	No. of thin melanoma patients with positive SLN	No. of patients with positive SLN and predictor	Unadusted Odds Ratio (95%CI)	Pooled Positive Rate (95%CI) (%)
**Breslow thickness <0.8mm**	23	23426	1228	469	–	2.9 (2.1–3.7)
**Breslow thickness ≥0.8mm**	23	23426	1228	759	1.61 (1.42–1.82)	7.0 (6.0–8.0)
**Ulceration**	25	17768	1108	115	1.60 (1.30–1.97)	4.2 (1.8–7.2)
**Regression**	14	11065	585	119	0.89 (0.72–1.11)	5.2 (2.9–8.1)
**Clark Level IV/V**	24	15198	803	421	1.68 (1.45–1.95)	6.6 (5.7–7.6)
**Mitosis Rate >0/mm^2^**	18	15002	801	584	2.22 (1.88–2.63)	7.7 (6.3–9.1)
**Tumor-infiltrating Lymphocytes**	5	1613	91	51	0.69 (0.43–1.10)	4.3 (2.5–6.5)
**Lymphovascular Invasion**	4	1973	119	6	2.39 (1.00–5.75)	12.9 (0–37.4)
**Microsatellites**	4	1411	77	5	3.80 (1.38–10.47)	16.6 (2.4–36.6)
**Vertical Growth Phase**	5	1821	112	91	2.76 (1.72–4.43)	8.1 (6.3–10.1)
**Primary Tumor Location (trunk vs others)**	20	17345	1025	432	1.10 (0.96–1.26)	6.2 (4.5–8.2)
**Primary Tumor Location (extremities vs others)**	20	17345	1025	457	0.98 (0.86–1.12)	6.4 (4.4–8.7)

The adjusted ORs of pathologic features are shown in **Table 3**. There were only 11 studies that had adjusted OR data that could be analyzed. Pathologic features that were available for adjusted analysis were limited as the presence of ulceration, Breslow thickness ≥0.8 mm, mitosis rate >0/mm^2^, Clark level IV/V, and the presence of regression. Breslow thickness ≥0.8 mm, presence of ulceration, mitosis rate >0/mm^2^ showed a significant association with SLN positivity in the adjusted analysis, while Clark level IV/V did not show a significant correlation with SLN positivity. Among these, the presence of ulceration was the strongest predictor of positive SLNB results in the adjusted analysis, with an adjusted OR of 2.75 (95%CI, 1.65 to 4.60).

**Table 3:** Pooled adjusted odds ratio of high-risk pathologic features.

Predictor	No. of studies	No. of thin melanoma patients undergoing SLNB	Adjusted Odds Ratio	95%CI
Ulceration	8	14003	2.75	1.65–4.60
Breslow thickness ≥0.8mm	10	19381	1.94	1.28–2.95
Mitosis Rate >0/mm^2^	8	12101	1.63	1.13–2.36
Clark Level IV/V	9	11924	1.24	0.84–1.84
Regression	7	9881	1.20	0.89–1.63

The associations between SLN positivity and the primary tumor location, the absence or presence of regression, LVI, or TIL were found with insufficient evidence.

## Discussion

It is critical to identify thin melanoma with a worse prognosis so that patients can be able to receive precise therapies. Researchers around the world have been interested in investigating an effective prediction for the prognosis of thin melanoma. Several pieces of research have been published in the past few years. This study is the most recent and most comprehensive meta-analysis to date. Compared with the previous meta-analysis, this study included 19 newly published research articles since 2015, reporting 26,308 patients in total who had a diagnosis of thin melanoma and underwent SLNB.

The pooled estimated positive rate of SLNB in thin melanoma in this study was 5.1%, with a 95% CI of 4.5% to 5.6%. This result is similar to those found in preexisting meta-analyses, which reported pooled positive rates of 5.6%, 4.5%, and 5.1% (3–5), but we got narrower confidence intervals. A 5% risk threshold is often used for surgeons suggesting to perform SLNB for a patient (37, 77). Generally, SLNB is offered to patients with primary melanoma with Breslow thickness ≥0.8 mm with additional risk factors. But different risk factors are recommended in different guidelines (10, 11). Therefore we analyzed the predictive value of multiple pathological and clinical features for the positive SLN.

In this study, we not only updated the predictive value of pathologic features explored in the previous meta-analysis but also paid attention to primary tumor location, which was reported to be correlated with a positive SLN (34). We yielded some different results. Ulceration, Clark level, and Breslow thickness were commonly recorded features in patients, reporting in 37.9%, 36.4%, and 34.8% of included studies, respectively. In the unadjusted analysis in our study, we recognized the same significant predictors as the previous meta-analysis and the primary tumor location was not significantly related to SLN positivity. And in the adjusted analysis in our study, however, the presence of ulceration was the most predictive factor for SLN positivity, while Clark level IV/V did not show a significant correlation with SLN positivity.

A limitation of the previous meta-analysis is the relatively small sample size of included studies. Only one study provided the data on the pathologic features of patients with a sample size larger than 1,000 for analysis. Several large-scale studies were published after 2015 which supplemented the insufficiency of the previous meta-analysis in the adjusted odds ratios analyses. In our study, 6 pieces of literature with a sample size larger than 1,000 were included. The largest one is the study of Conic, et al. published in 2019 with a sample size of 9,186, and it provided data on pathologic features that are available for both unadjusted and adjusted OR analyzing. Thus, we could obtain more accurate predictive values of pathologic and clinical features for SLN positivity. And the 95% CIs of unadjusted ORs for all features analyzed in our study were narrower than those reported in the previous meta-analysis.

The presence of microsatellites was recognized to have a 3.8-fold higher risk and positive rate of 16.6% for SLN positivity in our study, which means it is the strongest predictor among the pathologic features we analyzed. Microsatellites are a rarely present pathologic feature associated with poor prognosis and are more likely found in thicker melanoma (78). Four studies in our meta-analysis including 1411 patients with thin melanoma reported data on microsatellites (18, 25, 32, 41). Two of them demonstrated a remarkable increase in SLN positive rate when microsatellites were present, but none of the four studies found it statistically significant because of the infrequence of events. Adjusted analysis for microsatellites was not available because relevant researches were too few. And it is the same reason why the adjusted analysis was not done for the vertical growth phase. Regression in primary melanoma has been reported as a protective factor that relates to lower SLN positivity (79) and lower risk of death (80). A host immunologic response to the tumor is considered to play a role in the presence of regression. However, regression did not show significance relativity of SLN positivity in unadjusted analysis nor adjusted analysis in this study.

The pooled positive rate of SLNB in thin melanoma in this study was 5.1%. When patients were confirmed with melanomas of Breslow thickness ≥0.8 mm or mitosis rate >0/mm^2^, the pooled positive rate of SLNB would rise to 7.0% and 7.7%, respectively. Therefore, we suggest that surgeons should consider giving SLNB to such patients. And when a combination of high-risk features is found, the patient should be informed of the even higher rate of SLN positivity.

Our study has some limitations. All studies performed SLNB only in patients with thin melanoma when there was any high-risk feature; therefore, the overall positive rate of SLNB was undoubtedly higher than the true incidence of SLN positivity in all thin melanomas. Significant heterogeneity among the included studies (I^2 =^ 73.6%, p<0.001) was found using a weight estimated random-effects model in the meta-analysis. This probably resulted from several included studies with a higher proportion of positive SLNs. The reporting of identical pathologic features, such as mitosis rate, differed in some of the included studies by defining different cutoff values. This may lead to bias in analyzing its odds ratio. Since this meta-analysis was based on the study level, this variation could also increase the heterogeneity. A patient-level meta-analysis may help to avoid this variation and assess adjusted ORs for more pathologic features. For pathologic features such as microsatellites and the vertical growth phase, more research is needed to clarify their predictive value with larger data sets. Besides the risk factors analyzed in this study, there are other factors that affect the prognosis of melanoma. Melanin pigmentation plays a role in regulating melanocyte and neighboring cells’ behavior (81). It protects melanocytes from UVR but at times accelerates the progression of melanoma and makes melanocytes resistant to different types of therapy (82–84). And as a result, melanin pigmentation shortens overall survival and disease-free survival in metastatic melanoma (82). However, no study has reported the relationship between melanin pigmentation and a positive sentinel lymph node. We look forward to future researches.

## Conclusion

The overall positive rate of SLNB in thin melanoma in this study was 5.1%. The strongest predictor for SLN positivity identified was the presence of microsatellites on unadjusted analysis and the presence of ulceration on adjusted analysis. Breslow thickness ≥0.8 mm and mitosis rate >0/mm^2^ both predict SLN positivity in adjusted analysis and increase the positive rate to 7.0% and 7.7%. We suggest patients with thin melanoma with the above high-risk features should be considered for giving an SLNB.

## Data Availability Statement

The original contributions presented in the study are included in the article/supplementary material. Further inquiries can be directed to the corresponding authors.

## Author Contributions

JH and XL contributed to conception and design of the study. ZF and HH performed articles review and quality assessments. HH performed the data analyses and wrote the first draft of manuscript. JJ, ZF, and HH wrote sections of the manuscript. JJ, JH, and XL helped perform the analysis with constructive discussions. All authors contributed to manuscript revision, read, and approved the submitted version.

## Conflict of Interest

The authors declare that the research was conducted in the absence of any commercial or financial relationships that could be construed as a potential conflict of interest.

## Publisher’s Note

All claims expressed in this article are solely those of the authors and do not necessarily represent those of their affiliated organizations, or those of the publisher, the editors and the reviewers. Any product that may be evaluated in this article, or claim that may be made by its manufacturer, is not guaranteed or endorsed by the publisher.
